# Design of an acoustic superlens using single-phase metamaterials with a star-shaped lattice structure

**DOI:** 10.1038/s41598-018-19374-2

**Published:** 2018-01-30

**Authors:** Meng Chen, Heng Jiang, Han Zhang, Dongsheng Li, Yuren Wang

**Affiliations:** 1 Key Laboratory of Microgravity, Institute of Mechanics, Chinese Academy of Sciences, Beijing, 100190 China; 2 University of Chinese Academy of Sciences, Beijing, 100049 China; 3 State Key Laboratory of Acoustics, Institute of Acoustics, Chinese Academy of Sciences, 100190 Beijing, China; 4 National Key Laboratory on Ship Vibration & Noise, China Ship Scientific Research Center, Wuxi, 214082 China

## Abstract

We propose a single-phase super lens with a low density that can achieve focusing of sound beyond the diffraction limit. The super lens has a star-shaped lattice structure made of steel that offers abundant resonances to produce abnormal dispersive effects as determined by negative parameter indices. Our analysis of the metamaterial band structure suggests that these star-shaped metamaterials have double-negative index properties, that can mediate these effects for sound in water. Simulations verify the effective focusing of sound by a single-phase solid lens with a spatial resolution of approximately 0.39 *λ*. This superlens has a simple structure, low density and solid nature, which makes it more practical for application in water-based environments.

## Introduction

Achieving high-resolution super focusing of sound has been a longstanding challenge. The critical issue in solving super-resolution imaging centers around how to detect evanescent waves^1^, and this problem has been considerably ameliorated by the recent development of sonic metamaterials^2–5^. Sonic metamaterials are usually engineered in a complex fashion through subwavelength-scale resonant units to produce exotic physical properties through negative moduli^6,7^ and a negative mass density^8,9^. These properties enable the focusing of sound to overcome the diffraction limit according to the negative refraction and surface states^2^. Based on the super-resolution imaging approach offered by metamaterials^1^, a series of super lenses has been developed using a variety of sonic metamaterials with double-negative^10–13^, single-negative^14–17^ or near-zero mass properties^18,19^. However, their structures are usually complicated and bulky due to the need to construct the resonant units^20^. Super-lens design could therefore significantly benefit from the introduction of a new, simple and light resonant structure.

The traditional resonant structure of acoustic metamaterials used to design a super lens can be divided into four types: Helmholtz resonators^11,16,21^, three-component resonators^15,22^, holey-structured metamaterials^23–25^, and lumped mass structures^26–31^. Helmholtz resonators, which usually induce a negative modulus, were first used in the excogitation of a super lens and were designed as a planar network to focus ultrasound in water^11^. Subsequently, the design of a superlens with a negative effective mass was proposed based on a three-component metamaterial made of rubber-coated gold spheres in epoxy^15^. Similarly, a solid lens with hybrid resonators was produced that used negative refractive indices to focus waves^22^. Holey-structure metamaterials assembled from metal plates with drilled holes have been proposed to achieve super-resolution imaging due to their Fabry-Pérot (FP) resonance. However, they usually require the lens thickness to be equal to the integer number of the half-wavelength to satisfy the FP resonant condition. Finally, lumped mass structures, such as a perforated slab^26–29^, pillar structures^30^ or membrane-based structures^31^, usually composed of large pieces of solid material connected by small or soft connectors, can also be designed to act as a super lens due to their negative properties. However, the above-mentioned structures are all too complicated to use and often require the use of multiphase materials.

Lattice structures comprising an interconnected network of elastic beams, for examplethe Kagome lattice^32^, re-entrant grid^33^ and zigzag lattices^34,35^ structures, are widely used in standalone configurations due to their simple construction and low density. From the point of view of their wave characteristics, they have abundant bending resonators that more easily form a low-frequency band gap and exhibit extraordinary properties^32–36^. In this respect, they are the ideal structures for the design of new lightweight super-lenses; however, most of the current studies have focused on the band gap of lattice structures.

In this paper, we employed a lattice structure to design a solid super-lens for use in water due to its single-phase and lightweight construction. Our design process was focused on two design criteria. The first criterion was how to achieve the negative parameters required for a single-phase lattice structure. A dipole resonance system (lumped mass) is needed to generate a negative density (negative modulus) or a flexural resonance system (this calls for beams with a sufficiently large slenderness ratio)^37–40^. The second criterion centered on how to focus longitudinal sound (in a water environment) for the solid lens (coexisting with longitudinal and transverse modes)^22^. The structure needs to be prone to volumetric deformation and undergo strong coupling with sound in water. Based on the above considerations, a lattice structure with a star shape was proposed for the solid super lens. A star-shaped metamaterial has a special re-entrant structure and square-symmetrical configuration, which makes volumetric deformation easier^41,42^. When the slenderness ratio of the beams is large enough, it can produce an abundance of resonances that contain both dipole resonances and bending resonances, giving rise to abnormal dispersive effects associated with the beam’s negative parameters. The analysis presented below suggests that because of their abundant vibration modes, single-phase metamaterials with star-shaped structures result in double-negative properties with certain frequency bands and are ideal structures to construct solid super lenses.

## Results and Discussion

**Figure 1:**
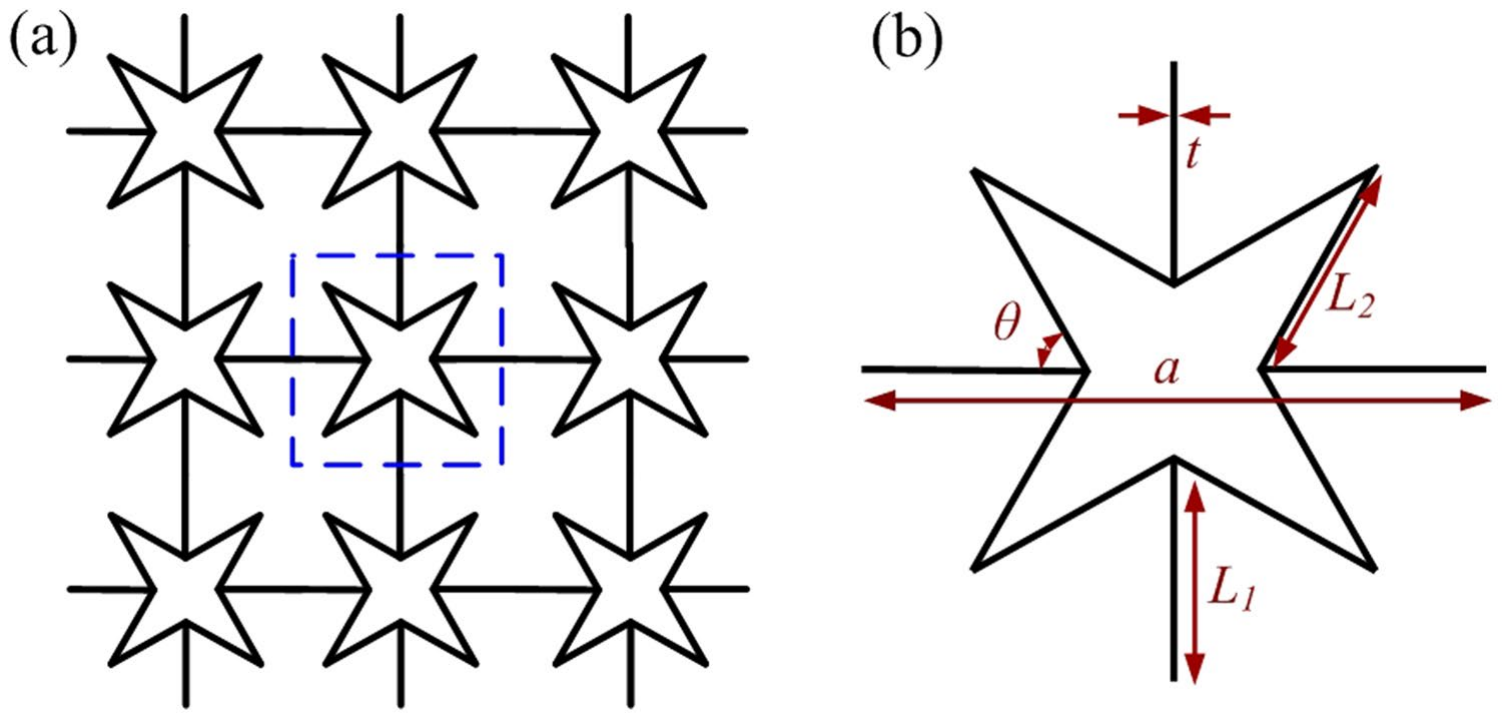
Structural layout (**a**) and unit cell (**b**) of the four-point star-shaped structure.

Star-shaped lattice structures have been widely studied due to their special property, negative Poisson’s ratio, and attractive mechanical and physical properties such as low weight, high strength, and high-energy absorption^42^. However, their wave manipulation characteristics, especially the effective parameter indices, have rarely been investigated. According to the cell-structure type, two-dimensional star-shaped structures can be classified as four-point or six-point structures, with the former being our focus of attention for the design of the super lens outlined here, see Fig. 1(a). This structure is a simple square lattice with a square unit cell, shown in Fig. 1(b), composed of four re-entrant corners of equal length *L*_2_ that are symmetrically arranged and joined by four straight beams of equal length *L*_1_ and thickness *t*. The corner angle between the adjacent cell walls is denoted by *θ*, and the lattice constant is denoted by *a*. The relationship between the geometric parameters is given by:

1 $$a=2\times \{\frac{sin(\alpha -45^\circ )}{sin(45^\circ )}\cdot {L}_{2}+{L}_{1}\}$$

The porosity can be calculated as:

2 $$f=1-\frac{4\times ({L}_{1}+2\times {L}_{2})\times t}{{a}^{2}}$$

Our structural parameter settings are: straight rib length *L*_1_ = 1 cm, re-entrant corner length *L*_2_ = 1 cm, thickness *t* = 0.05 cm, corner angle *θ* = 70°, and lattice constant *a* = 2.793 cm. The corresponding porosity of the star-shaped lattice structure is calculated to be 0.923. Although the entire structure is made of steel, it is more lightweight than other traditional structures. The values of steel’s material parameters are density *ρ* = 7.78 g/cm^3^, Young’s modulus *E* = 210.6 GPa, and shear modulus *G* = 81 MPa. The band structure and effective parameters of the star-shaped structure are analyzed next and are used to design the negative-index solid super-lens.

**Figure 2:**
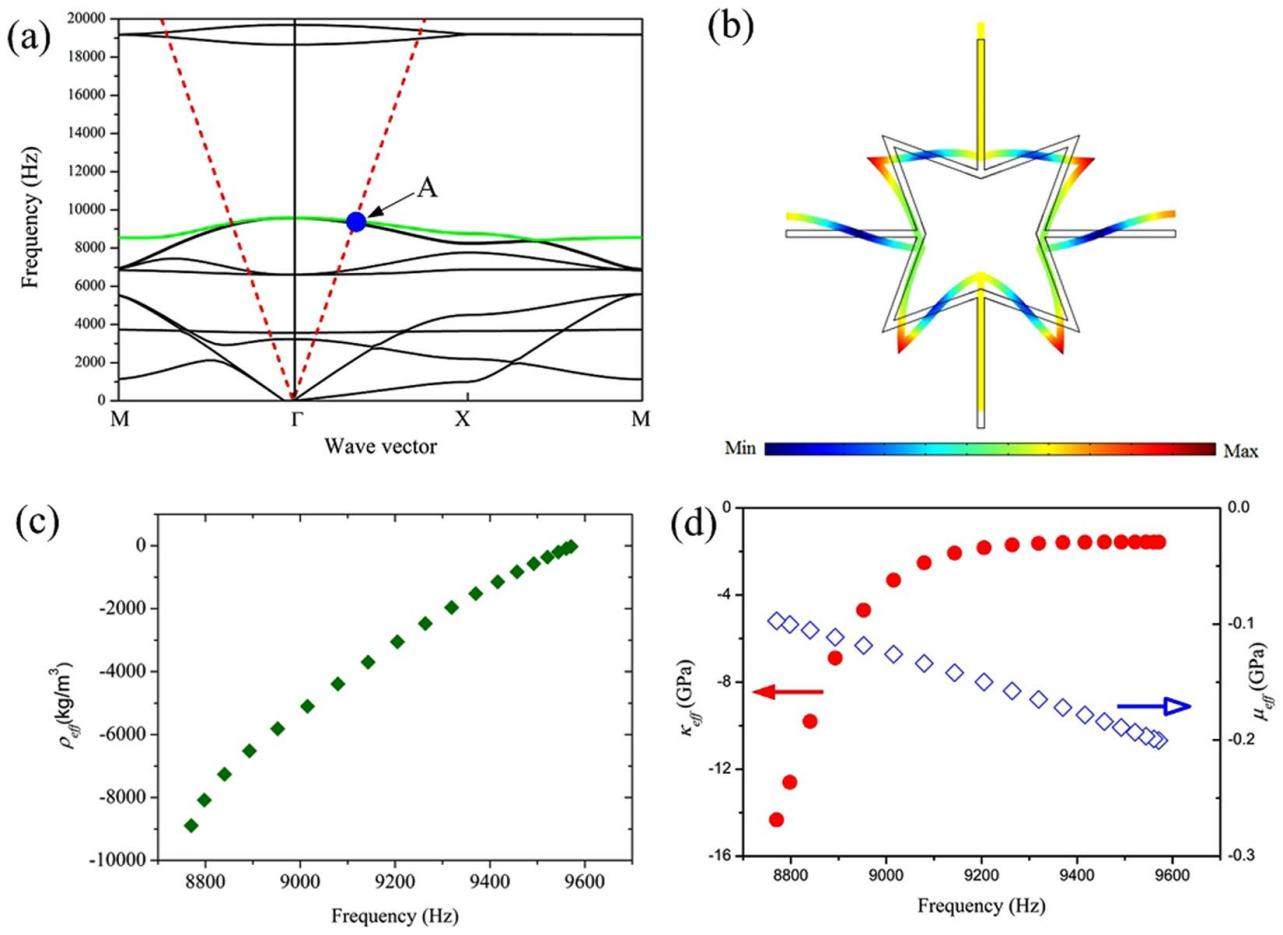
(**a**) Band structure of a four-point star-shaped structure of the infinite crystal (solid lines) and the dispersion curves of water (dashed red line). (**b**) Displacement distributions of the unit cell corresponding to the eigenstate marked “A” in part (**a**). (**c**,**d**) Calculated effective mass density *ρ*_*eff*_, effective bulk *κ*_*eff*_ and shear moduli *μ*_*eff*_ as a function of frequency.

The band structure along the MΓXM path of the irreducible Brillouin zone of the simple square lattice (Fig. 2a) shows two low-frequency band gaps at 5591–6610 Hz and 9574–18653 Hz. Negative slopes are observed in the seventh and eighth branches in the frequency band 8760–9574 Hz, where the refractive indices of the star-shaped structure are negative according to metamaterials theory^37,39^. The eighth branch, shown by the green line in Fig. 2(a), is our central focus as it corresponds to a longitudinal mode, which couples easily to incident longitudinal sound waves in water^22^. The distribution of the displacements of the unit cell, Fig. 2(b), corresponding to eigenmode “A”, Fig. 2(a), indicates that the deformation mainly involves the bending of the slender beams and hence differs from that of traditional sonic metamaterials. This bending mode can be decomposed into a translational motion along the vertical straight beams and bending of the other beams. The translational motion can be seen as a mass-spring system: the entire eight cant beams act as a lumped mass while the four straight beams act as a spring. Moreover, the translational motion of the beams is similar to the vibration mode of the three-component metamaterial at the lower edge of the band gap, as for a dipole vibration, which can produce a negative effective mass density. Meanwhile, the bending of the other beams is equivalent to the rotational deformation of the four points of the star, which provides the negative effective modulus. These two modes constitute a hybrid state that has double-negative index properties in this specific frequency band. The double-negative properties of star-shaped lattice structures can be achieved by a hybrid state that consists of a dipole resonance from translation of the lumped mass and a rotating state arising from bending.

To verify the double-negative index properties of this star-shaped structure, the effective parameter values were calculated. With a lattice constant of approximately 2.8 cm, the wavelength is approximately six times that of the lattice constant in the frequency band 8760–9574 Hz. From effective-medium theory, wave behaviors are described by effective parameters that can be obtained using the surface integration method^39,40^. Both the effective mass density and modulus of the eighth branch along the ΓX direction, as shown in Fig. 2(c) and (d) respectively, show that the effective density *ρ*_*eff*_, bulk modulus *κ*_*eff*_, and shear modulus *μ*_*eff*_ are all negative in this frequency band. Compared to traditional sonic metamaterials, the star-shaped metamaterial has a simple structure composed of a single-phase material. Note that the values of the effective density *ρ*_*eff*_ and bulk modulus *κ*_*eff*_ are close to that of water for frequencies from 9300 Hz to 9430 Hz, which is of interest for developing a super lens.

**Figure 3:**
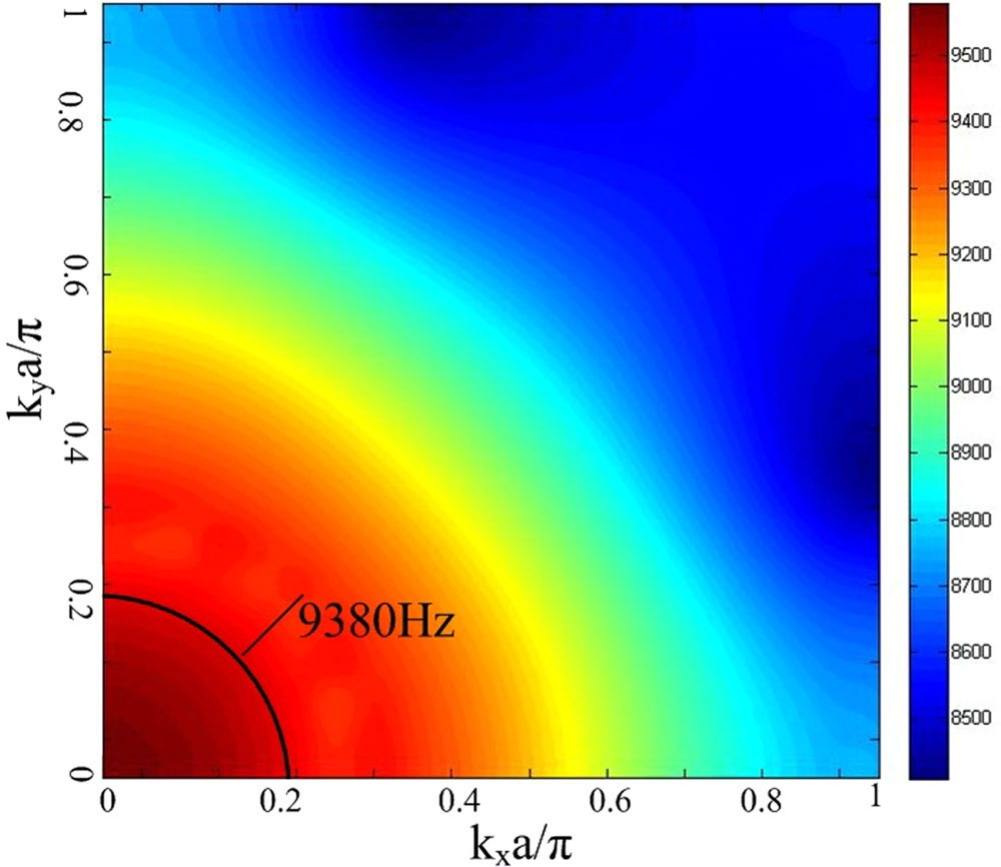
Eigenfrequency contour plot of a sonic metamaterial with a star-shaped structure.

The dispersion curve of sound in water, indicated by the red dashed line in Fig. 2(a), intersects the negative branch at two slightly different frequencies, specifically 9409 Hz along the ΓX direction and 9311 Hz along the ΓM direction, which implies that waves near these frequencies will focus in water. We further checked the equifrequency contour at these frequencies, as shown in Fig. 3, which appears to be nearly circular at 9380 Hz. Thus all of the angles of the star-shaped structure metamaterial have an effective negative refractive index of approximately minus one relative to water, which is the condition required for sound convergence^24^. The results suggest that the star-shaped structure will behave as a super lens near a frequency of 9380 Hz.

**Figure 4:**
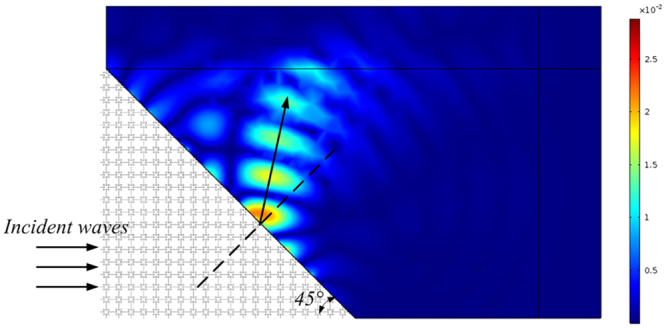
Pressure-field distribution obtained fromthe negative refraction simulation with a Gaussian pulse at the central frequency of 9380 Hz.

To test the negative refraction of the star-shaped lattice structure, a 45° wedged sample consisting of 210 unit cells with a square array in a water environment was simulated, with the resulting pressure-field distribution shown in Fig. 4. A Gaussian acoustic pressure beam with a central frequency of 9380 Hz was launched in the fluid from the left side of the wedge. Figure 4 shows that the incident wave and refracted wave are located on both sides of the normal, which suggests that the energy flux of the refraction wave outside the sample travels on the negative-refraction side at this frequency. This typical negative refraction phenomenon further substantiates the fact that the star-shaped lattice structure, which simultaneously possesses a negative effective modulus and negative effective density induced by the hybrid state, exhibits both translational and bending resonances.

**Figure 5:**
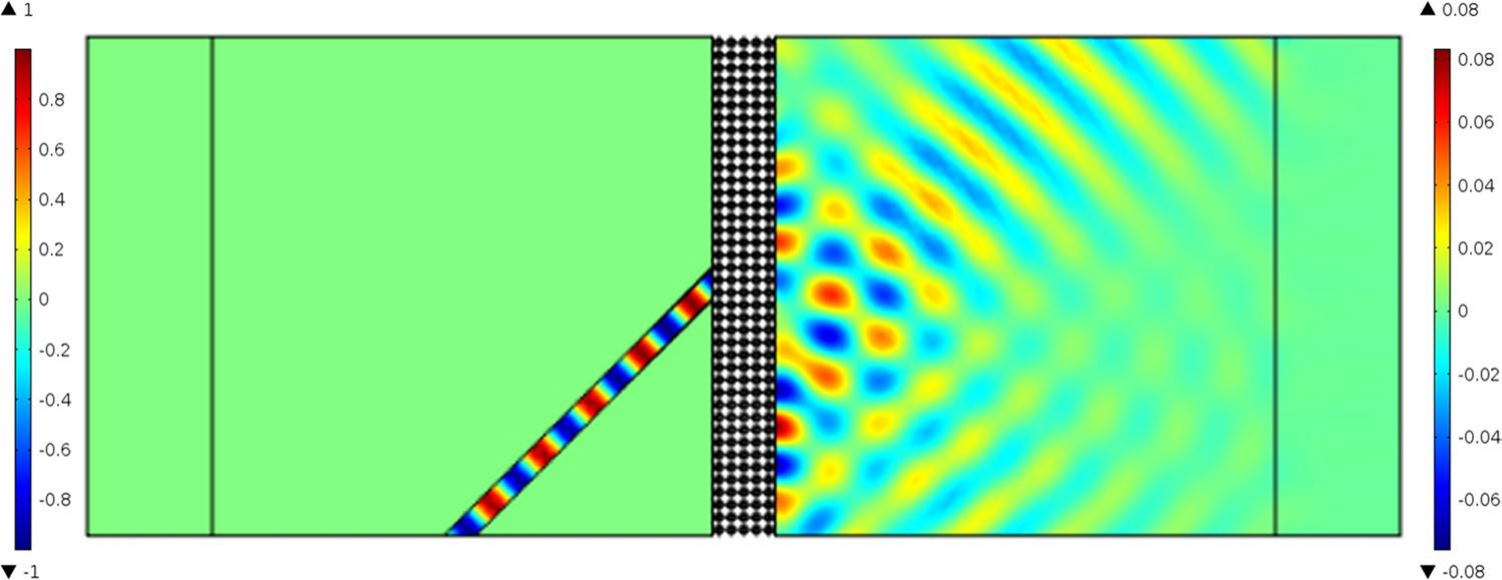
Pressure-field distribution obtained from the negative refraction simulation with a plane wave at 9380 Hz.

To obtain super-focusing, the first requirement is a negative refractive index material. To determine whether this requirement was met, we considered a 9380-Hz plane wave incident on a finite slab with five star-shaped unit cells from the left (Fig. 5). The plane wave makes an angle of 45° to the vertical axis, incident at the center of a 5-cm-wide slab. Both the incident and transmitted waves are located on the left-hand side of the vertical axis, and the transmitted waves follow the inverted Snell law in the star-shaped structure. During propagation, the wave beams are negatively refracted twice. According to the location of the incident and transmitted waves, the negative refractive index was calculated and found to be close to minus one. Simulations were also performed to determine the negative refractive properties of the star-shaped metamaterial.

**Figure 6:**
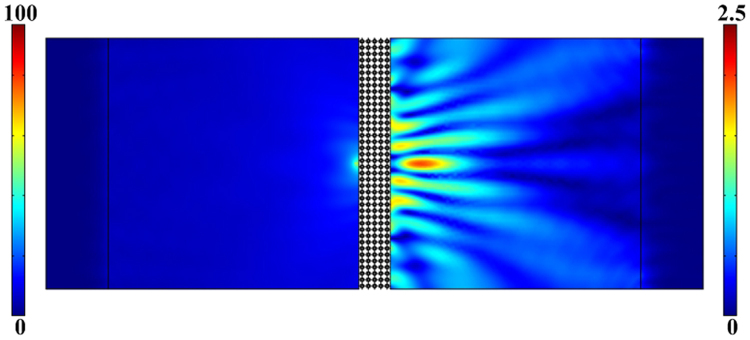
Pressure-field distribution obtained in a simulation of focusing with a point wave-source with a frequency of 9380 Hz.

From these simulations, a super lens was established with a star-shaped lattice structure, and its focusing capability was examined theoretically. The super-lens was five unit cells thick (~14 cm thick, or one wavelength in water at 9380 Hz), and 50 unit cells wide (~140 cm thick, sufficient to avoid edge effects). The whole structure was immersed in water in the simulation. To produce super-resolution focusing, a point wave source at 9380 Hz excited the surface of the lens, approximately 2.8 cm away from the surface. The pressure-field distribution around the slab reveals an image spot that is clearly observable behind the lens (Fig. 6). Because the point source was located close to the lens surface, the location of the image was approximately 13.4 cm behind the lens, and therefore the focusing distance, *i*, was approximately equal to the lens thickness, in accordance with acoustic ray tracing theories^22^. Note that the amplitude of the image spot was far less than that of the source. This is because there was an effective impedance mismatch between the star-shaped lens and water. Optimizing the structure and materials could improve the impedance matching.

**Figure 7:**
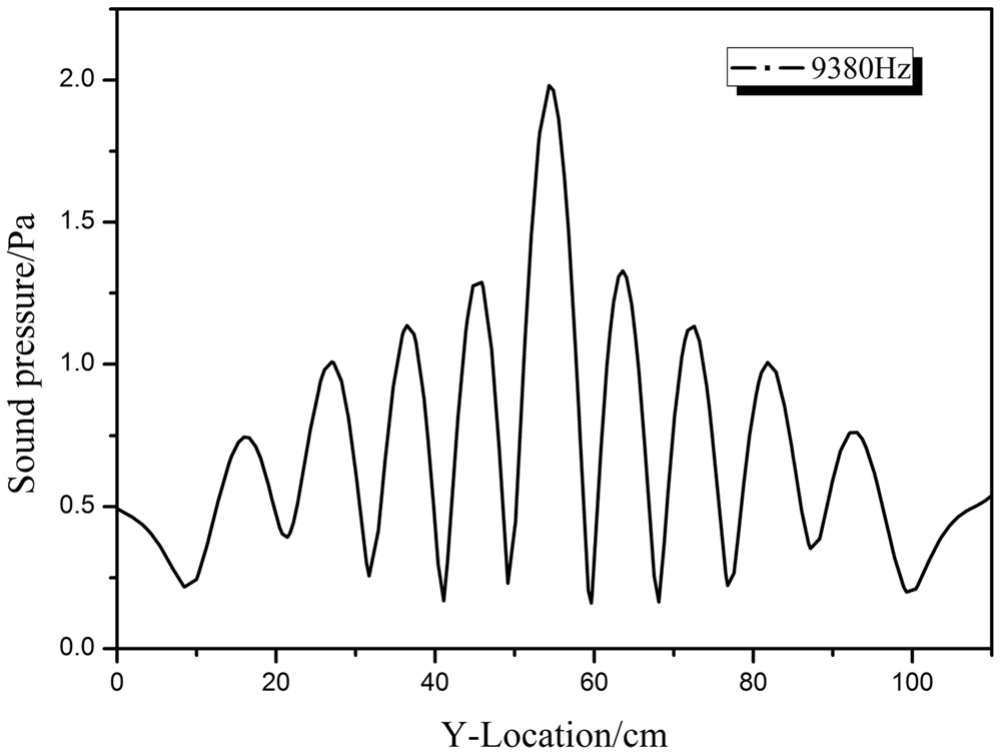
Pressure intensity of the focusing spot (*i* ≈ 13.4 cm) behind the lens.

To determine the spatial resolution of the lens, we calculated the pressure intensity along the line through the focusing spot parallel to the lens surface (Fig. 7). The lateral resolution was estimated from the full-width-at-half-maximum intensity of the transmission peak, according to the Rayleigh criterion. The spatial resolution of the lens with the star-shaped structure was approximately 0.39 *λ* (6.3 cm) at 9380 Hz, which was less than the resolution limit of 0.5 *λ*. This shows that the single-phase, solid lens with a simple star-shaped structure is able to achieve focusing of sound beyond the diffraction limit. Its impedance requires further examination.

## Conclusion

We propose the use of a star-shaped Metamaterial for constructing an acoustic superlens in water. The metamaterial’s band structure and effective parameter values suggest it exhibits double-negative index properties in the frequency band from 8760–9574 Hz, where its refractive indices are negative. Eigenfrequency-contour and negative-refraction simulations further confirm that the refractive index of the star-shaped structure is approximately minus one at 9380 Hz, which implies that the lens is able to focus sound waves at this frequency. Numerical simulations reveal that the lens offers a spatial resolution of 0.39 *λ*, less than the diffraction limit of 0.5 *λ*.

With its simple geometry, this super lens is easier to implement in practice. Moreover, its solid nature makes it more suitable for use in water environments. The results suggest that this novel acoustic super-lens design could open up new, practical applications of superlenses based on metamaterials.

## Model and Methods

The finite element method (FEM) was used to calculate the band structure and effective medium parameters of our sonic metamaterial^39,40^. A distinct advantage of the FEM is its flexibility in modeling complex structures made of various materials, good convergence, and high precision. Employing the FEM software *COMSOL Multiphysics* to calculate the band structure and design the super-lens, the calculation model was reduced to a single unit by applying Bloch boundary conditions on opposing boundaries. The entire band structure was obtained by sweeping the wave vector along the edges of the irreducible Brillouin zone.

A unit cell with a complex structure can be considered to be a basic element unit that responds to external stimulation as a whole under the long-wavelength assumption. The effective parameter values of the medium were calculated by determining the displacement, strain, stress, and force on the boundaries^39,40^. During calculations, periodic boundary conditions were introduced so that an infinite structure could be realized by the unit cell. In addition, to secure the local fields, time-harmonic displacement was applied on one boundary of the unit cell before computation.

The effective mass was calculated according to Newton’s second law:

3 $${\rho }^{eff}=\frac{{m}^{eff}}{{a}^{2}}=\frac{{F}_{x}^{eff}}{{\ddot{u}}_{x}^{eff}{a}^{2}}=\frac{=-{F}_{x}^{eff}}{{\omega }^{2}{u}_{x}^{eff}{a}^{2}}$$

where *ρ*^*eff*^ is the effective density, ${F}_{x}^{eff}$ is the effective net force exerted on the unit cell in the *x*-direction, and ${u}_{x}^{eff}$ is the effective displacement of the unit cell in the *x*-direction. ${F}_{x}^{eff}$ and ${u}_{x}^{eff}$ can be obtained as follows:

4 $${F}_{x}^{eff}\int {T}_{xx}{dy|}_{x=a}-\int {T}_{xx}{dy|}_{x=0}+\int {T}_{xy}{dx|}_{y=a}-\int {T}_{xy}{dx|}_{y=0}$$

5 $${u}_{x}^{eff}=\frac{\int {{u}_{x}dy|}_{x=0}+\int {{u}_{x}dy|}_{x=a}}{2a}$$

The moduli were calculated using constitutive relations:

6 $$\begin{array}{c}{T}_{xx}^{eff}={C}_{11}^{eff}{S}_{xx}^{eff}+{C}_{12}^{eff}{S}_{yy}^{eff}\\ {T}_{yy}^{eff}={C}_{12}^{eff}{S}_{xx}^{eff}+{C}_{11}^{eff}{S}_{yy}^{eff}\\ {T}_{xy}^{eff}=2{C}_{44}^{eff}{S}_{xy}^{eff}\end{array}$$

Here ${C}_{11}^{eff}$, ${C}_{12}^{eff}$, and ${C}_{44}^{eff}$ are the effective stiffness tensors; ${T}_{xx}^{eff}$, ${T}_{yy}^{eff}$, and ${T}_{xy}^{eff}$ are the xx, yy, and xy components of the effective stress tensor, respectively; ${S}_{xx}^{eff}$, ${S}_{yy}^{eff}$, and ${S}_{xy}^{eff}$ are the *xx, yy*, and *xy* components of the effective strain tensor, respectively.

Under external stimulation, there are three unknowns in the constitutive relations: ${C}_{11}^{eff}$, ${C}_{12}^{eff}$, and ${C}_{44}^{eff}$. The others can be obtained from the stress and deformations of the unit boundary.

${T}_{xx}^{eff}$, ${T}_{yy}^{eff}$, and ${T}_{xy}^{eff}$ were calculated as follows:

7 $$\begin{array}{c}{T}_{xx}^{eff}=\frac{\int {{T}_{xx}dy|}_{x=0}+\int {{T}_{xx}dy|}_{x=a}}{2a}\\ {T}_{yy}^{eff}=\frac{\int {{T}_{yy}dx|}_{y=0}+\int {{T}_{yy}dx|}_{y=a}}{2a}\\ {T}_{xy}^{eff}=\frac{\int {{T}_{xy}dx|}_{y=0}+\int {{T}_{xy}dx|}_{y=a}}{2a}\end{array}$$

${S}_{xx}^{eff}$, ${S}_{yy}^{eff}$, and ${S}_{xy}^{eff}$ were calculated as follows:

8 $$\begin{array}{c}{S}_{xx}^{eff}=\frac{\int {{u}_{x}dy|}_{x=a}-\int {{u}_{x}dy|}_{x=0}}{{a}^{2}}\\ {S}_{yy}^{eff}=\frac{\int {{u}_{y}dy|}_{x=a}-\int {{u}_{y}dy|}_{x=0}}{{a}^{2}}\\ {S}_{xy}^{eff}=\frac{\int {{u}_{x}dx|}_{y=a}-\int {{u}_{x}dx|}_{y=0}+\int {{u}_{y}dy|}_{x=a}-\int {{u}_{y}dy|}_{x=0}}{2{a}^{2}}\end{array}$$

The effective bulk modulus ${\kappa }^{eff}$ and effective shear modulus *μ*^*eff*^ were defined by ${C}_{11}^{eff}$, ${C}_{12}^{eff}$, and ${C}_{44}^{eff}$:

9 $${\kappa }^{eff}=\frac{{c}_{11}^{eff}+{c}_{12}^{eff}}{2}$$

10 $${\mu }^{eff}=\frac{{c}_{11}^{eff}-{c}_{12}^{eff}}{2}$$

To verify the negative refractive index properties of the star-shaped lattice structure, a 45° prism-shaped structure consisting of 210 unit cells in a water environment was simulated (as shown in Fig. 5). Using the time domain module in *COMSOL Multiphysics*, a Gaussian pulse with a central frequency of 9380 Hz in an open domain was launched from the left, vertically incident to the interface between the steel structure and water. The negative-refraction (shown in Fig. 6) and sound-focusing simulations (Fig. 7) were carried out in the frequency domain by calculating the transmission response using the finite period model. A wide slab consisting of 5×20 unit cells in a water environment was established. Meanwhile, to reduce the reflected wave at the boundary, a perfect matching layer was used to characterize the infinite medium. The mass density and sound velocity of water were set as 1000 kg/m^3^ and 1480 m/s.
